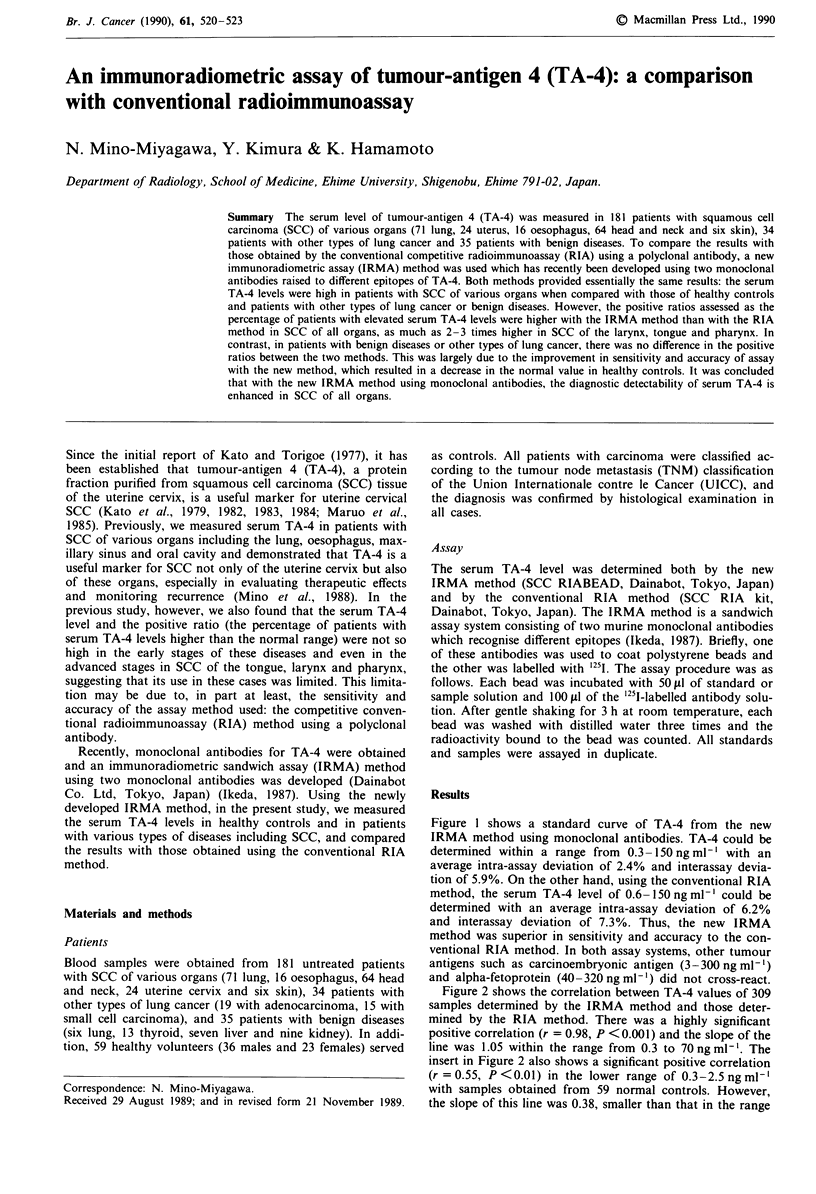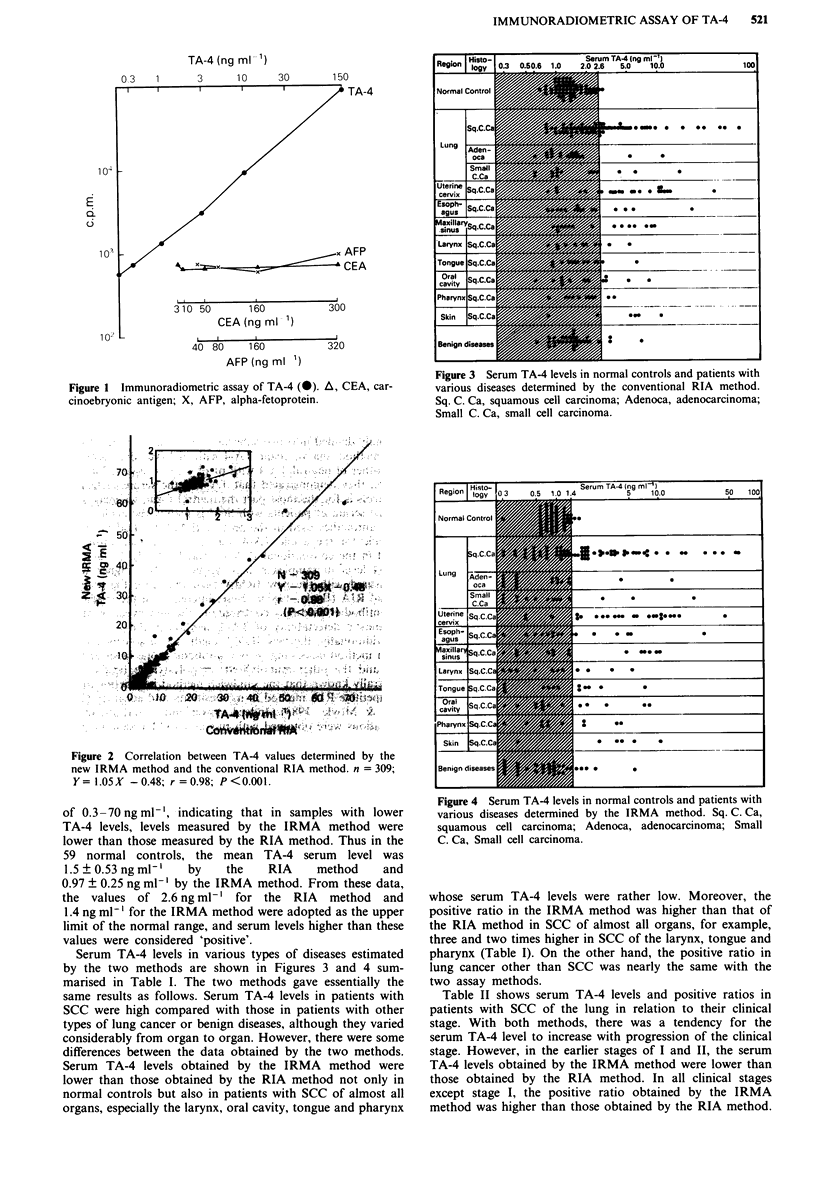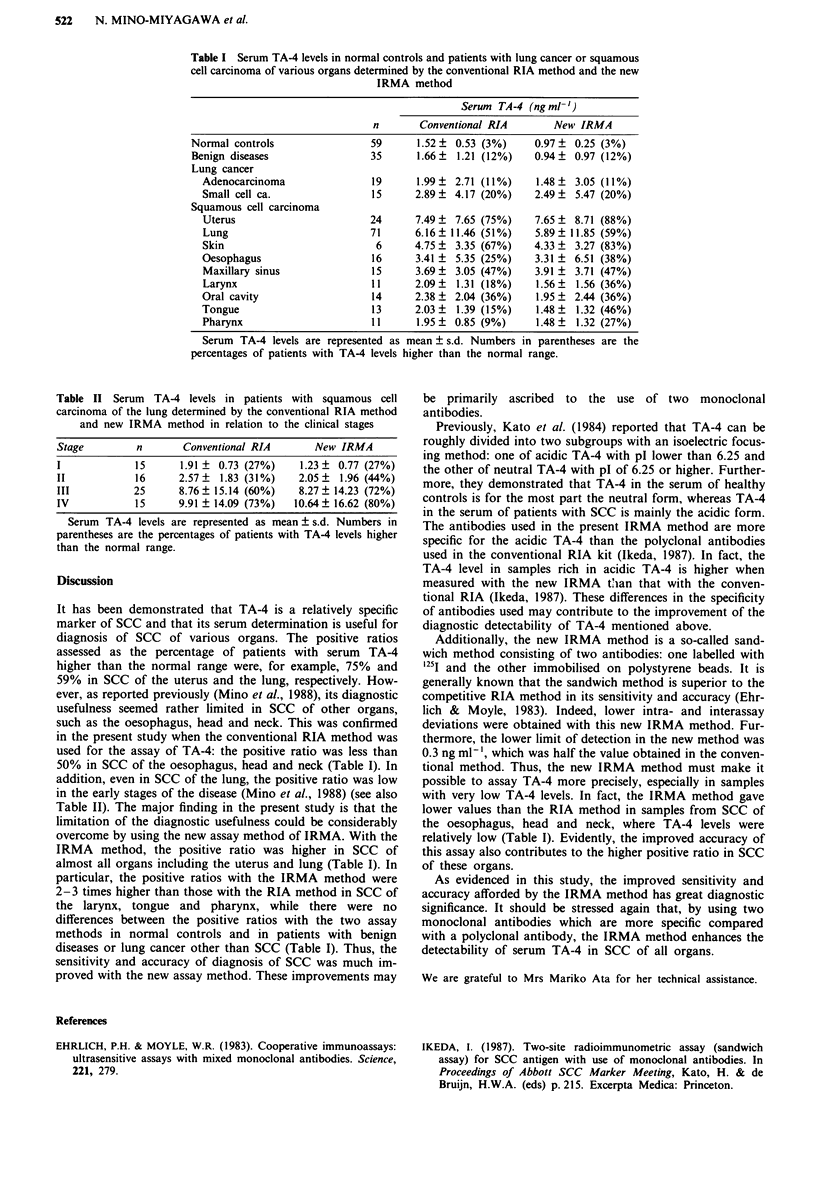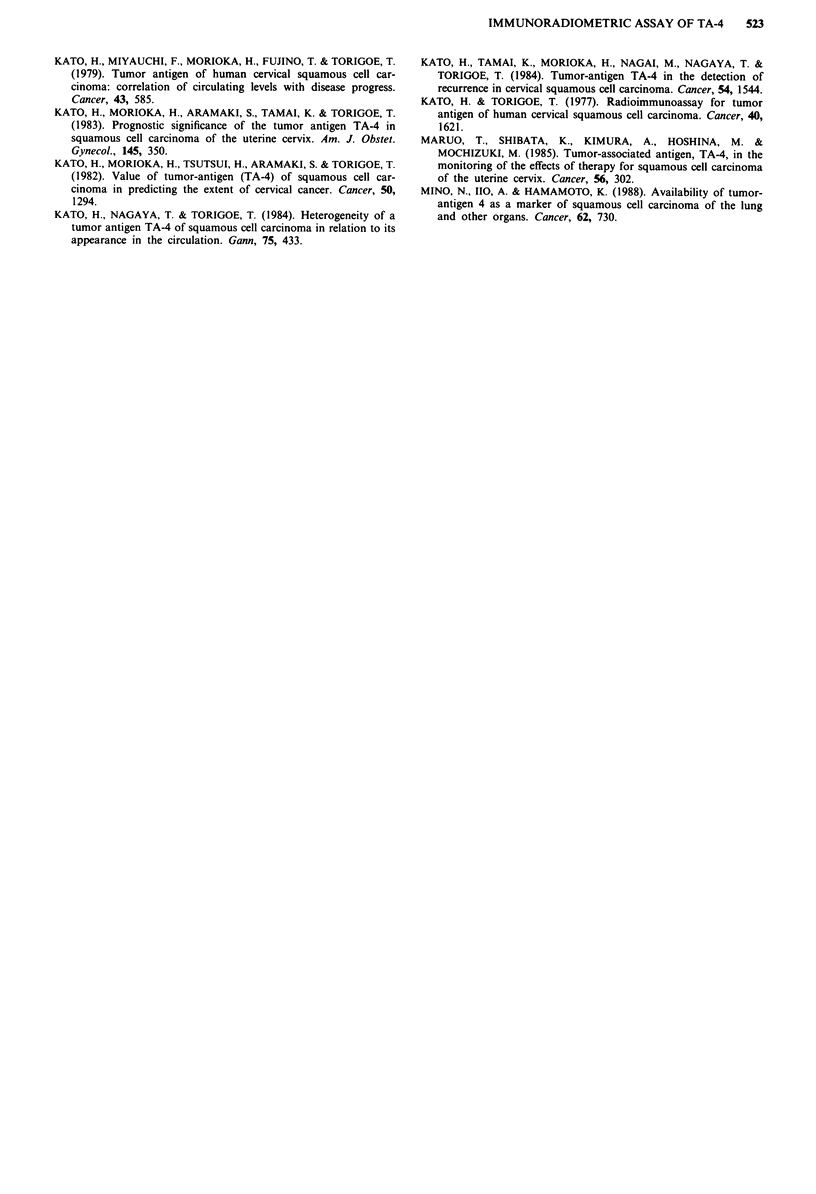# An immunoradiometric assay of tumour-antigen 4 (TA-4): a comparison with conventional radioimmunoassay.

**DOI:** 10.1038/bjc.1990.117

**Published:** 1990-04

**Authors:** N. Mino-Miyagawa, Y. Kimura, K. Hamamoto

**Affiliations:** Department of Radiology, School of Medicine, Ehime University, Japan.

## Abstract

The serum level of tumour-antigen 4 (TA-4) was measured in 181 patients with squamous cell carcinoma (SCC) of various organs (71 lung, 24 uterus, 16 oesophagus, 64 head and neck and six skin), 34 patients with other types of lung cancer and 35 patients with benign diseases. To compare the results with those obtained by the conventional competitive radioimmunoassay (RIA) using a polyclonal antibody, a new immunoradiometric assay (IRMA) method was used which has recently been developed using two monoclonal antibodies raised to different epitopes of TA-4. Both methods provided essentially the same results: the serum TA-4 levels were high in patients with SCC of various organs when compared with those of healthy controls and patients with other types of lung cancer or benign diseases. However, the positive ratios assessed as the percentage of patients with elevated serum TA-4 levels were higher with the IRMA method than with the RIA method in SCC of all organs, as much as 2-3 times higher in SCC of the larynx, tongue and pharynx. In contrast, in patients with benign diseases or other types of lung cancer, there was no difference in the positive ratios between the two methods. This was largely due to the improvement in sensitivity and accuracy of assay with the new method, which resulted in a decrease in the normal value in healthy controls. It was concluded that with the new IRMA method using monoclonal antibodies, the diagnostic detectability of serum TA-4 is enhanced in SCC of all organs.


Br. J. Cancer (1990), 61, 520-523 © Macmillan Press Ltd., 1990

### An immunoradiometric assay of tumour-antigen 4 (TA-4): a comparison with conventional radioimmunoassay

N. Mino-Miyagawa, Y. Kimura & K. Hamamoto

*Department of Radiology, School of Medicine, Ehime University, Shigenobu, Ehime 791-02, Japan.*

**Summary** The serum level of tumour-antigen 4 (TA-4) was measured in 181 patients with squamous cell carcinoma (SCC) of various organs (71 lung, 24 uterus, 16 oesophagus, 64 head and neck and six skin), 34 patients with other types of lung cancer and 35 patients with benign diseases. To compare the results with those obtained by the conventional competitive radioimmunoassay (RIA) using a polyclonal antibody, a new immunoradiometric assay (IRMA) method was used which has recently been developed using two monoclonal antibodies raised to different epitopes of TA-4. Both methods provided essentially the same results: the serum TA-4 levels were high in patients with SCC of various organs when compared with those of healthy controls and patients with other types of lung cancer or benign diseases. However, the positive ratios assessed as the percentage of patients with elevated serum TA-4 levels were higher with the IRMA method than with the RIA method in SCC of all organs, as much as 2-3 times higher in SCC of the larynx, tongue and pharynx. In contrast, in patients with benign diseases or other types of lung cancer, there was no difference in the positive ratios between the two methods. This was largely due to the improvement in sensitivity and accuracy of assay with the new method, which resulted in a decrease in the normal value in healthy controls. It was concluded that with the new IRMA method using monoclonal antibodies, the diagnostic detectability of serum TA-4 is enhanced in SCC of all organs.

Since the initial report of Kato and Torigoe (1977), it has been established that tumour-antigen 4 (TA-4), a protein fraction purified from squamous cell carcinoma (SCC) tissue of the uterine cervix, is a useful marker for uterine cervical SCC (Kato *et al.*, 1979, 1982, 1983, 1984; Maruo *et al.*, 1985). Previously, we measured serum TA-4 in patients with SCC of various organs including the lung, oesophagus, maxillary sinus and oral cavity and demonstrated that TA-4 is a useful marker for SCC not only of the uterine cervix but also of these organs, especially in evaluating therapeutic effects and monitoring recurrence (Mino *et al.*, 1988). In the previous study, however, we also found that the serum TA-4 level and the positive ratio (the percentage of patients with serum TA-4 levels higher than the normal range) were not so high in the early stages of these diseases and even in the advanced stages in SCC of the tongue, larynx and pharynx, suggesting that its use in these cases was limited. This limitation may be due to, in part at least, the sensitivity and accuracy of the assay method used: the competitive conventional radioimmunoassay (RIA) method using a polyclonal antibody.

Recently, monoclonal antibodies for TA-4 were obtained and an immunoradiometric sandwich assay (IRMA) method using two monoclonal antibodies was developed (Dainabot Co. Ltd, Tokyo, Japan) (Ikeda, 1987). Using the newly developed IRMA method, in the present study, we measured the serum TA-4 levels in healthy controls and in patients with various types of diseases including SCC, and compared the results with those obtained using the conventional RIA method.

### Materials and methods

### *Patients*

Blood samples were obtained from 181 untreated patients with SCC of various organs (71 lung, 16 oesophagus, 64 head and neck, 24 uterine cervix and six skin), 34 patients with other types of lung cancer (19 with adenocarcinoma, 15 with small cell carcinoma), and 35 patients with benign diseases (six lung, 13 thyroid, seven liver and nine kidney). In addition, 59 healthy volunteers (36 males and 23 females) served as controls. All patients with carcinoma were classified according to the tumour node metastasis (TNM) classification of the Union Internationale contre le Cancer (UICC), and the diagnosis was confirmed by histological examination in all cases.

### *Assay*

The serum TA-4 level was determined both by the new IRMA method (SCC RIABEAD, Dainabot, Tokyo, Japan) and by the conventional RIA method (SCC RIA kit, Dainabot, Tokyo, Japan). The IRMA method is a sandwich assay system consisting of two murine monoclonal antibodies which recognise different epitopes (Ikeda, 1987). Briefly, one of these antibodies was used to coat polystyrene beads and the other was labelled with $^{125}$I. The assay procedure was as follows. Each bead was incubated with 50 $\mu$l of standard or sample solution and 100 $\mu$l of the $^{125}$I-labelled antibody solution. After gentle shaking for 3 h at room temperature, each bead was washed with distilled water three times and the radioactivity bound to the bead was counted. All standards and samples were assayed in duplicate.

### Results

Figure 1 shows a standard curve of TA-4 from the new IRMA method using monoclonal antibodies. TA-4 could be determined within a range from 0.3-150 ng ml$^{-1}$ with an average intra-assay deviation of 2.4% and interassay deviation of 5.9%. On the other hand, using the conventional RIA method, the serum TA-4 level of 0.6-150 ng ml$^{-1}$ could be determined with an average intra-assay deviation of 6.2% and interassay deviation of 7.3%. Thus, the new IRMA method was superior in sensitivity and accuracy to the conventional RIA method. In both assay systems, other tumour antigens such as carcinoembryonic antigen (3-300 ng ml$^{-1}$) and alpha-fetoprotein (40-320 ng ml$^{-1}$) did not cross-react.

Figure 2 shows the correlation between TA-4 values of 309 samples determined by the IRMA method and those determined by the RIA method. There was a highly significant positive correlation ($r = 0.98$, $P < 0.001$) and the slope of the line was 1.05 within the range from 0.3 to 70 ng ml$^{-1}$. The insert in Figure 2 also shows a significant positive correlation ($r = 0.55$, $P < 0.01$) in the lower range of 0.3-2.5 ng ml$^{-1}$ with samples obtained from 59 normal controls. However, the slope of this line was 0.38, smaller than that in the range

Correspondence: N. Mino-Miyagawa.
Received 29 August 1989; and in revised form 21 November 1989.

**Figure 1** Immunoradiometric assay of TA-4 (●). Δ, CEA, carcinoebryonic antigen; X, AFP, alpha-fetoprotein.

**Figure 2** Correlation between TA-4 values determined by the new IRMA method and the conventional RIA method. $n = 309$; $Y = 1.05X - 0.48$; $r = 0.98$; $P < 0.001$.

**Figure 3** Serum TA-4 levels in normal controls and patients with various diseases determined by the conventional RIA method. Sq. C. Ca, squamous cell carcinoma; Adenoca, adenocarcinoma; Small C. Ca, small cell carcinoma.

**Figure 4** Serum TA-4 levels in normal controls and patients with various diseases determined by the IRMA method. Sq. C. Ca, squamous cell carcinoma; Adenoca, adenocarcinoma; Small C. Ca, Small cell carcinoma.

of 0.3–70 ng $ml^{-1}$, indicating that in samples with lower TA-4 levels, levels measured by the IRMA method were lower than those measured by the RIA method. Thus in the 59 normal controls, the mean TA-4 serum level was $1.5 \pm 0.53$ ng $ml^{-1}$ by the RIA method and $0.97 \pm 0.25$ ng $ml^{-1}$ by the IRMA method. From these data, the values of 2.6 ng $ml^{-1}$ for the RIA method and 1.4 ng $ml^{-1}$ for the IRMA method were adopted as the upper limit of the normal range, and serum levels higher than these values were considered 'positive'.

Serum TA-4 levels in various types of diseases estimated by the two methods are shown in Figures 3 and 4 summarised in Table I. The two methods gave essentially the same results as follows. Serum TA-4 levels in patients with SCC were high compared with those in patients with other types of lung cancer or benign diseases, although they varied considerably from organ to organ. However, there were some differences between the data obtained by the two methods. Serum TA-4 levels obtained by the IRMA method were lower than those obtained by the RIA method not only in normal controls but also in patients with SCC of almost all organs, especially the larynx, oral cavity, tongue and pharynx whose serum TA-4 levels were rather low. Moreover, the positive ratio in the IRMA method was higher than that of the RIA method in SCC of almost all organs, for example, three and two times higher in SCC of the larynx, tongue and pharynx (Table I). On the other hand, the positive ratio in lung cancer other than SCC was nearly the same with the two assay methods.

Table II shows serum TA-4 levels and positive ratios in patients with SCC of the lung in relation to their clinical stage. With both methods, there was a tendency for the serum TA-4 level to increase with progression of the clinical stage. However, in the earlier stages of I and II, the serum TA-4 levels obtained by the IRMA method were lower than those obtained by the RIA method. In all clinical stages except stage I, the positive ratio obtained by the IRMA method was higher than those obtained by the RIA method.

**Table I** Serum TA-4 levels in normal controls and patients with lung cancer or squamous cell carcinoma of various organs determined by the conventional RIA method and the new IRMA method

| | | Serum TA-4 ($ng\,ml^{-1}$) | |
|---|---|---|---|
| | n | Conventional RIA | New IRMA |
| Normal controls | 59 | 1.52 ± 0.53 (3%) | 0.97 ± 0.25 (3%) |
| Benign diseases | 35 | 1.66 ± 1.21 (12%) | 0.94 ± 0.97 (12%) |
| Lung cancer | | | |
| Adenocarcinoma | 19 | 1.99 ± 2.71 (11%) | 1.48 ± 3.05 (11%) |
| Small cell ca. | 15 | 2.89 ± 4.17 (20%) | 2.49 ± 5.47 (20%) |
| Squamous cell carcinoma | | | |
| Uterus | 24 | 7.49 ± 7.65 (75%) | 7.65 ± 8.71 (88%) |
| Lung | 71 | 6.16 ± 11.46 (51%) | 5.89 ± 11.85 (59%) |
| Skin | 6 | 4.75 ± 3.35 (67%) | 4.33 ± 3.27 (83%) |
| Oesophagus | 16 | 3.41 ± 5.35 (25%) | 3.31 ± 6.51 (38%) |
| Maxillary sinus | 15 | 3.69 ± 3.05 (47%) | 3.91 ± 3.71 (47%) |
| Larynx | 11 | 2.09 ± 1.31 (18%) | 1.56 ± 1.56 (36%) |
| Oral cavity | 14 | 2.38 ± 2.04 (36%) | 1.95 ± 2.44 (36%) |
| Tongue | 13 | 2.03 ± 1.39 (15%) | 1.48 ± 1.32 (46%) |
| Pharynx | 11 | 1.95 ± 0.85 (9%) | 1.48 ± 1.32 (27%) |

Serum TA-4 levels are represented as mean ± s.d. Numbers in parentheses are the percentages of patients with TA-4 levels higher than the normal range.

**Table II** Serum TA-4 levels in patients with squamous cell carcinoma of the lung determined by the conventional RIA method and new IRMA method in relation to the clinical stages

| Stage | n | Conventional RIA | New IRMA |
|---|---|---|---|
| I | 15 | 1.91 ± 0.73 (27%) | 1.23 ± 0.77 (27%) |
| II | 16 | 2.57 ± 1.83 (31%) | 2.05 ± 1.96 (44%) |
| III | 25 | 8.76 ± 15.14 (60%) | 8.27 ± 14.23 (72%) |
| IV | 15 | 9.91 ± 14.09 (73%) | 10.64 ± 16.62 (80%) |

Serum TA-4 levels are represented as mean ± s.d. Numbers in parentheses are the percentages of patients with TA-4 levels higher than the normal range.

### Discussion

It has been demonstrated that TA-4 is a relatively specific marker of SCC and that its serum determination is useful for diagnosis of SCC of various organs. The positive ratios assessed as the percentage of patients with serum TA-4 higher than the normal range were, for example, 75% and 59% in SCC of the uterus and the lung, respectively. However, as reported previously (Mino *et al.*, 1988), its diagnostic usefulness seemed rather limited in SCC of other organs, such as the oesophagus, head and neck. This was confirmed in the present study when the conventional RIA method was used for the assay of TA-4: the positive ratio was less than 50% in SCC of the oesophagus, head and neck (Table I). In addition, even in SCC of the lung, the positive ratio was low in the early stages of the disease (Mino *et al.*, 1988) (see also Table II). The major finding in the present study is that the limitation of the diagnostic usefulness could be considerably overcome by using the new assay method of IRMA. With the IRMA method, the positive ratio was higher in SCC of almost all organs including the uterus and lung (Table I). In particular, the positive ratios with the IRMA method were 2–3 times higher than those with the RIA method in SCC of the larynx, tongue and pharynx, while there were no differences between the positive ratios with the two assay methods in normal controls and in patients with benign diseases or lung cancer other than SCC (Table I). Thus, the sensitivity and accuracy of diagnosis of SCC was much improved with the new assay method. These improvements may be primarily ascribed to the use of two monoclonal antibodies.

Previously, Kato *et al.* (1984) reported that TA-4 can be roughly divided into two subgroups with an isoelectric focusing method: one of acidic TA-4 with pI lower than 6.25 and the other of neutral TA-4 with pI of 6.25 or higher. Furthermore, they demonstrated that TA-4 in the serum of healthy controls is for the most part the neutral form, whereas TA-4 in the serum of patients with SCC is mainly the acidic form. The antibodies used in the present IRMA method are more specific for the acidic TA-4 than the polyclonal antibodies used in the conventional RIA kit (Ikeda, 1987). In fact, the TA-4 level in samples rich in acidic TA-4 is higher when measured with the new IRMA than that with the conventional RIA (Ikeda, 1987). These differences in the specificity of antibodies used may contribute to the improvement of the diagnostic detectability of TA-4 mentioned above.

Additionally, the new IRMA method is a so-called sandwich method consisting of two antibodies: one labelled with $^{125}I$ and the other immobilised on polystyrene beads. It is generally known that the sandwich method is superior to the competitive RIA method in its sensitivity and accuracy (Ehrlich & Moyle, 1983). Indeed, lower intra- and interassay deviations were obtained with this new IRMA method. Furthermore, the lower limit of detection in the new method was 0.3 $ng\,ml^{-1}$, which was half the value obtained in the conventional method. Thus, the new IRMA method must make it possible to assay TA-4 more precisely, especially in samples with very low TA-4 levels. In fact, the IRMA method gave lower values than the RIA method in samples from SCC of the oesophagus, head and neck, where TA-4 levels were relatively low (Table I). Evidently, the improved accuracy of this assay also contributes to the higher positive ratio in SCC of these organs.

As evidenced in this study, the improved sensitivity and accuracy afforded by the IRMA method has great diagnostic significance. It should be stressed again that, by using two monoclonal antibodies which are more specific compared with a polyclonal antibody, the IRMA method enhances the detectability of serum TA-4 in SCC of all organs.

We are grateful to Mrs Mariko Ata for her technical assistance.

### References

EHRLICH, P.H. & MOYLE, W.R. (1983). Cooperative immunoassays: ultrasensitive assays with mixed monoclonal antibodies. *Science*, **221**, 279.

IKEDA, I. (1987). Two-site radioimmunometric assay (sandwich assay) for SCC antigen with use of monoclonal antibodies. In *Proceedings of Abbott SCC Marker Meeting*, Kato, H. & de Bruijn, H.W.A. (eds) p. 215. Excerpta Medica: Princeton.

KATO, H., MIYAUCHI, F., MORIOKA, H., FUJINO, T. & TORIGOE, T. (1979). Tumor antigen of human cervical squamous cell carcinoma: correlation of circulating levels with disease progress. *Cancer*, **43**, 585.

KATO, H., MORIOKA, H., ARAMAKI, S., TAMAI, K. & TORIGOE, T. (1983). Prognostic significance of the tumor antigen TA-4 in squamous cell carcinoma of the uterine cervix. *Am. J. Obstet. Gynecol.*, **145**, 350.

KATO, H., MORIOKA, H., TSUTSUI, H., ARAMAKI, S. & TORIGOE, T. (1982). Value of tumor-antigen (TA-4) of squamous cell carcinoma in predicting the extent of cervical cancer. *Cancer*, **50**, 1294.

KATO, H., NAGAYA, T. & TORIGOE, T. (1984). Heterogeneity of a tumor antigen TA-4 of squamous cell carcinoma in relation to its appearance in the circulation. *Gann*, **75**, 433.

KATO, H., TAMAI, K., MORIOKA, H., NAGAI, M., NAGAYA, T. & TORIGOE, T. (1984). Tumor-antigen TA-4 in the detection of recurrence in cervical squamous cell carcinoma. *Cancer*, **54**, 1544.

KATO, H. & TORIGOE, T. (1977). Radioimmunoassay for tumor antigen of human cervical squamous cell carcinoma. *Cancer*, **40**, 1621.

MARUO, T., SHIBATA, K., KIMURA, A., HOSHINA, M. & MOCHIZUKI, M. (1985). Tumor-associated antigen, TA-4, in the monitoring of the effects of therapy for squamous cell carcinoma of the uterine cervix. *Cancer*, **56**, 302.

MINO, N., IIO, A. & HAMAMOTO, K. (1988). Availability of tumor-antigen 4 as a marker of squamous cell carcinoma of the lung and other organs. *Cancer*, **62**, 730.